# Synergistic effects of free radical scavengers and cochlear vasodilators: a new otoprotective strategy for age-related hearing loss

**DOI:** 10.3389/fnagi.2015.00086

**Published:** 2015-05-15

**Authors:** Juan Carlos Alvarado, Verónica Fuentes-Santamaría, Pedro Melgar-Rojas, María Llanos Valero, María Cruz Gabaldón-Ull, Josef M. Miller, José M. Juiz

**Affiliations:** ^1^Facultad de Medicina, Universidad de Castilla-La Mancha, Instituto de Investigación en Discapacidades Neurológicas (IDINE)Albacete, Spain; ^2^Karolinska InstitutetStockholm, Sweden; ^3^Kresge Hearing Research Institute, University of MichiganAnn Arbor, MI, USA

**Keywords:** antioxidants, vitamins, cochlear blood flow, magnesium, oxidative stress, presbyacusis, sensorineural hearing loss

## Abstract

The growing increase in age-related hearing loss (ARHL), with its dramatic reduction in quality of life and significant increase in health care costs, is a catalyst to develop new therapeutic strategies to prevent or reduce this aging-associated condition. In this regard, there is extensive evidence that excessive free radical formation along with diminished cochlear blood flow are essential factors involved in mechanisms of other stress-related hearing loss, such as that associated with noise or ototoxic drug exposure. The emerging view is that both play key roles in ARHL pathogenesis. Therapeutic targeting of excessive free radical formation and cochlear blood flow regulation may be a useful strategy to prevent onset of ARHL. Supporting this idea, micronutrient-based therapies, in particular those combining antioxidants and vasodilators like magnesium (Mg^2+^), have proven effective in reducing the impact of noise and ototoxic drugs in the inner ear, therefore improving auditory function. In this review, the synergistic effects of combinations of antioxidant free radicals scavengers and cochlear vasodilators will be discussed as a feasible therapeutic approach for the treatment of ARHL.

## Introduction

Despite the fact that age-related hearing loss (ARHL) affects more than one-third of the world population over 60 years-old, rising to more than two-third of those in their 70’s (Ohlemiller and Frisina, 2008; Gopinath et al., 2009; Lin et al., 2011; Yamasoba et al., 2013), currently there is no available medical treatment for this age-related sensory dysfunction. This has led to an important humanitarian cost in terms of isolation, frustration, depression, cognitive decline and decrease in quality of life (World Health Organization, 2002, 2013; Huang and Tang, 2010; Kidd III and Bao, 2012; Ciorba et al., 2012), along with an enormous and growing economic burden in health care costs (World Health Organization, 2002, 2013; Huang and Tang, 2010). In an attempt to address this issue, recent research has focused on understanding the cellular mechanisms that participate in the development and progression of ARHL, in order to refine its diagnosis and facilitate the design of new therapeutic strategies to prevent or reduce this sensory impairment and it consequences. Animal models have been valuable tools for the evaluation of this complex and multifactorial condition; and have provided significant information on the underlying genetic, molecular, histological and physiological factors associated with ARHL (Syka, 2002, 2010; Ohlemiller, 2006; Bielefeld et al., 2008, 2010; Fetoni et al., 2011; Alvarado et al., 2014). Previous studies have demonstrated that similar to that which occurs in other stress-related auditory pathologies, such as noise and drug-induced hearing loss (Ames et al., 1993; Ohlemiller, 2006; Chen et al., 2009; Bielefeld et al., 2010; Huang and Tang, 2010; Fetoni et al., 2011; Haider et al., 2014), an excess of free radical formation and blood flow reduction in the cochlea may be critical factors in triggering hearing loss associated with aging (Seidman et al., 2002; Bielefeld et al., 2010; Fetoni et al., 2011; Fujimoto and Yamasoba, 2014).

## Free Radical Formation and Blood Flow Reduction in Cochlea

As part of normal cellular homeostasis, free radicals, notably reactive oxygen species (ROS), are continuously generated during aerobic respiration as by-products of redox reactions, mostly in mitochondria (Ames et al., 1993; Chen et al., 2009; Bielefeld et al., 2010; Huang and Tang, 2010; Fujimoto and Yamasoba, 2014). Free radicals are unstable molecular species that contain one or more unpaired electrons, which make them highly reactive (Halliwell, 2006; Halliwell and Gutteridge, 2007). It is noteworthy, that although all oxygen radicals are ROS, not all ROS are oxygen radicals, leading researchers to distinguish between oxygen non-radical species and reactive radical/non-radical species (e.g., reactive nitrogen species, reactive bromide species, and reactive chlorine species) (for a detailed summary of ROS, see Halliwell, 2006; Halliwell and Gutteridge, 2007). Under normal conditions, adequate intracellular ROS levels are essential to regulate many cell signaling pathways (Finkel, 2012; Ray et al., 2012; Sena and Chandel, 2012) and cellular homeostasis (Sena and Chandel, 2012), among other cellular functions. However, as a consequence of imbalances in production of free radicals and endogenous antioxidant systems, ROS concentrations may increase, become toxic, and cause oxidative stress-induced cell damage (Ames et al., 1993; Halliwell, 2006; Halliwell and Gutteridge, 2007; Chen et al., 2009; Sena and Chandel, 2012; Böttger and Schacht, 2013; Fujimoto and Yamasoba, 2014). As ROS-induced reactions proceed, other excessive free radicals, such as nitric monoxide, peroxide, superoxide, hydroxyl or peroxyl radicals (Ames et al., 1993; Seidman et al., 2002; Halliwell, 2006; Halliwell and Gutteridge, 2007; Uttara et al., 2009; Park and Yeo, 2013; Fujimoto and Yamasoba, 2014), interact causing oxidative damage of lipids and proteins in cell membranes and the cytosol, mitochondrial and nuclear genome mutations, and ultimately lead to cellular death (Ames et al., 1993; Uttara et al., 2009; Lee and Wei, 2012; Fujimoto and Yamasoba, 2014).

**Figure 1:**
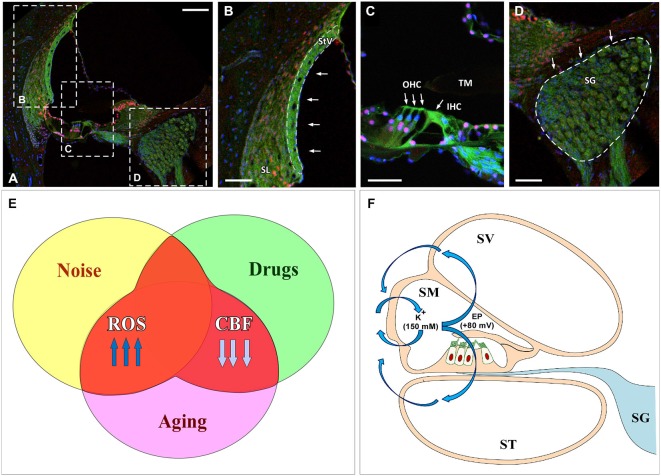
**Cochlear damage results in imbalances in free radical formation and cochlear blood supply in the inner ear following noise overstimulation, ototoxic drugs and aging. (A–D)** Confocal images show target cochlear structures affected by oxidative stress and reduced CBF: the StV and SL **(B)**, outer and inner hair cells (arrows in **C**) and the SG **(D)**. Dashed line in **(B)** outlines the StV. Filamentous actin was stained with Phalloidin (green) and cell nuclei with caspase (red) and DAPI (blue). **(E)** Excessive ROS along with reduced CBF lead to oxidative stress-induced cell damage causing disruption of the inner ear structure and function. **(F)** Injury to the stria vascularis induces a dysregulation of the EP (blue arrows) that affects K^(+)^ concentration, which in addition to diminished CBF, results in sensory epithelium disruption. Abbreviations: ROS, reactive oxygen species; CBF, cochlear blood flow; EP, endocochlear potential; SV, scala vestibuli; SM, scala media; ST, scala tympani; StV, stria vascularis; SL, spiral ligament; SG, spiral ganglion; TM, tectorial membrane; OHC, outer hair cells; IHC inner hair cell. Scale bars: 100 μm in **(A)**; 50 μm in **(B–D)**.

As postulated for several neurodegenerative diseases such as amyotrophic lateral sclerosis, Alzheimer’s, Parkinson’s and Huntington’s diseases (Ames et al., 1993; Lin and Beal, 2006), the cascade of molecular events related to ROS overproduction may play a crucial role during the aging process (Ames et al., 1993; Ohlemiller, 2006; Chen et al., 2009; Bielefeld et al., 2010; Huang and Tang, 2010; Fetoni et al., 2011; Fujimoto and Yamasoba, 2014; Haider et al., 2014; Ortuño-Sahagún et al., 2014). Specifically, an excess of free radicals in the cochlear sensory epithelium, spiral ganglion neurons and cells of the stria vascularis may have a relevant role in the development of ARHL (Figures 1A–D; Ohlemiller, 2006; Chen et al., 2009; Bielefeld et al., 2010; Huang and Tang, 2010; Fetoni et al., 2011; Fujimoto and Yamasoba, 2014). Of importance, excessive ROS build up is clearly the key factor in the pathogenesis of other stress-induced otological conditions that also result in reduced auditory function, such as noise and drug induced hearing loss (Figure 1E; Ohinata et al., 2003; Le Prell et al., 2007a,b, 2014; Bielefeld et al., 2010; Fetoni et al., 2011). These findings provide the rationale to support the hypothesis that therapeutic strategies targeting ROS overproduction may be potentially useful not only for ameliorating noise and drug induced hearing loss but also to improve ARLH. Thus we propose that excessive free radical formation may provide a “common pathogenic pathway”, shared by these pathologies (Figure 1E).

In addition to free radical generation in the cochlea, reduction in cochlear blood flow and vascular conductance during aging is another main contributor to cochlear damage. Consistent with this notion, during senescence there is a significant decrease in the circulating blood volume with reductions that may reach up to 20% in the cerebral flow (Park and Yeo, 2013). Despite the strong autoregulation of cochlear blood flow that occurs under normal conditions, the cochlea is no exception to this rule, as a significant decrease in blood flow regulation as well as in blood supply to the cochlea occurs during aging, particularly in the stria vascularis, in a number of animal models and man (Johnsson and Hawkins, 1972; Schuknecht and Gacek, 1993; Nakashima, 1999; Seidman et al., 1999; Seidman, 2000; Shi, 2011). Age-related alterations in the microvasculature of the stria vascularis, virtually the only vascularized epithelium in the body, have been found to correlate with the increase in auditory thresholds observed in presbyacusis, a condition known as strial or “metabolic” presbyacusis (Schuknecht and Gacek, 1993), as well as in noise and drug induced hearing loss, (Boettcher, 2002; Bielefeld et al., 2010; Fetoni et al., 2011; Shi, 2011; Lee, 2013; Ruan et al., 2014). The stria vascularis is pivotal in maintaining the endocochlear potential (EP; Figure 1F) as alterations in its structure and function induce a progressive decrease in the EP, finally affecting the cochlear amplification of acoustic signals (Gates and Mills, 2005; Schmiedt, 2010). Thus, diminished cochlear blood flow may contribute to damage to the stria vascularis and altered hair cell function (with or without cell death) and to aging-related increases in auditory thresholds (Shi, 2011; Lee, 2013). It is worth noting that recent evidence shows that there is a significant involvement of strial presbyacusis in the genesis of the ARHL, leading to the suggestion that alterations in the stria vascularis could be the major cause of hearing loss during aging (Schuknecht and Gacek, 1993; Gates and Mills, 2005; Schmiedt, 2010; Clinkard et al., 2013; Lee, 2013). In line with these observations, pharmacological up-regulation of cochlear blood flow could provide a vital treatment for ARHL.

## Free Radical Scavengers and Vasodilators

**Figure 2:**
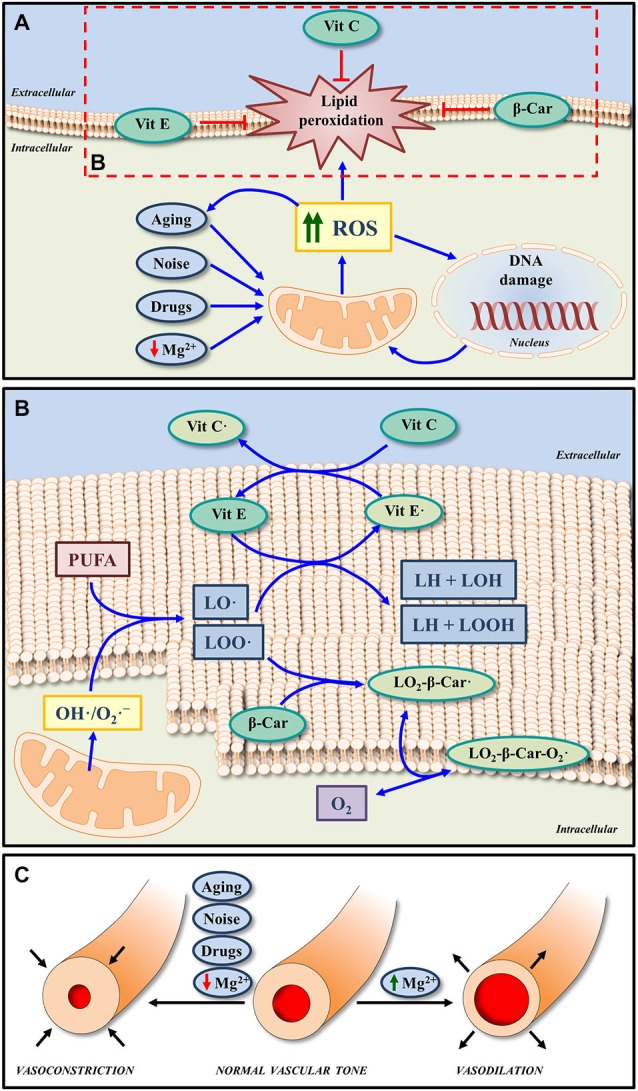
**Effects of micronutrients on oxidative stress and cochlea vasculature. (A)** Increased ROS generation induced by noise, drugs, aging and even low extracellular Mg^2+^, may lead to oxidative DNA damage and lipid peroxidation in the cell membrane. The non-enzymatic antioxidant system, which is composed by micronutrients such as Vit E, Vit C, and β-Car (metabolized to form Vit A) can block and/or revert lipid peroxidation, by reducing the impact of oxidative stress. **(B)** In response to ROS overproduction, peroxyl radicals of lipids (LOO·) and alkoxyl radicals of lipids (LO·) can be generated from polyunsaturated fatty acids (PUFA) of the cell membrane phospholipids. Both radicals can be scavenged by Vit E, one of the major antioxidants in the cell membrane. On the other hand, Vit C, considered one of the most important antioxidant molecules in the extracellular fluid, in addition to scavenging ROS, it can also protect cell membranes by regenerating Vit E from the oxidized form (Vit E·). Thus, the functions of both Vit E and Vit C in lipid peroxidation are coupled. Finally, the antioxidant activity of β-Car may also contribute to protect membranes from lipid peroxidation by scavenging LOO·. The reaction product (LO_2_-β-Car·) can react in turn with an oxygen molecule to generate a new peroxyl radical (LO_2_-β-Car-O_2_·). **(C)** A byproduct of free radical formation in the inner ear (8-Iso-Prostaglandin F_2_α) is a powerful vasoconstrictor; and thus reduced blood flow is found with intense noise exposure, which may be blocked by an isoprostane blocker or a cochlear vasodilator, such as Mg^2+^. Similarly Mg^2+^ will increase inner ear blood flow in the vascularly compromised aging ear. Abbreviations: ROS, reactive oxygen species; Vit A, vitamin A; Vit E, vitamin E; Vit C, vitamin C; β-Car, β-Carotene; Mg^2+^, magnesium; O_2_**·-**, superoxide radical; OH**·**, hydroxyl radicals.

In the cell, there are different and overlapping antioxidant systems of defense against oxidative stress. The enzymatic systems involved include superoxide dismutase, glutathione peroxidase, glutathione reductase and catalases while the non-enzymatic scavengers are vitamins and micronutrients (Figures 2A,B; Halliwell, 2006; Halliwell and Gutteridge, 2007). As an excess of free radical formation is likely involved in the pathogenesis of many types of hearing loss, the administration of antioxidants has been used to minimize or avoid inner ear damage in conditions such as noise and drug induced hearing loss (Yamasoba et al., 1999; Seidman et al., 2002; Ohinata et al., 2003; Yamashita et al., 2005; Le Prell et al., 2007a, 2011; Fetoni et al., 2011). Although there is still controversy about the benefits of using free radical scavengers for the treatment of ROS induced cochlear damage (Uttara et al., 2009; Bielefeld et al., 2010; Park and Yeo, 2013), most studies seem to agree that antioxidants reduce structural and functional stress-induced pathology in the inner ear in experimental animals (Yamasoba et al., 1999; Ohinata et al., 2003; Yamashita et al., 2005; Le Prell et al., 2007b; Bielefeld et al., 2010; Fetoni et al., 2011). For instance, mannitol (Yamasoba et al., 1999), N-acetylcysteine (Kopke et al., 2005, 2007), acetyl-L-carnitine (Kopke et al., 2005), sallicylates combined with N-acetylcysteine (Kopke et al., 2000, 2005), trolox (Yamashita et al., 2005) or vitamins A, C, and E (Le Prell et al., 2007a, 2011) attenuate inner ear damage following noise-induce hearing loss. Similarly, D-methionine (Campbell et al., 2007), N-acetylcysteine (Tokgoz et al., 2011) or a combination of vitamins A, C, and E (Le Prell et al., 2014) also have been shown to protect the cochlea after drug ototoxicity.

This growing body of evidence supports the use of antioxidants to ameliorate pathological related to excess ROS. A clear example of this is the prevention and interruption of ROS-induced lipid peroxidation in cell membranes by vitamins A, C, and E (Figures 2A,B; Halliwell, 2006; Halliwell and Gutteridge, 2007). Given the key role of ROS in ARHL (Ohlemiller, 2006; Chen et al., 2009; Bielefeld et al., 2010; Huang and Tang, 2010; Fetoni et al., 2011; Fujimoto and Yamasoba, 2014), it is likely that free radical scavengers may provide a pharmacological approach to treat presbyacusis. Supporting this expectation the administration of resveratrol (Seidman et al., 2003) or L-carnitine (Derin et al., 2004) in Fischer 344 and Wistar rats respectively, or a combination of L-cysteine-glutathione mixed disulfide, ribose-cysteine, NW-nitro-L-arginine methyl ester, vitamin B12, folate, and ascorbic acid in C57BL/6 mice (Heman-Ackah et al., 2010) led to improved auditory function and delayed onset of ARHL. In possible contradiction, the administration of either vitamin C in senescent marker protein 30/gluconolactonase knockout mice, which cannot synthesize vitamin C (Kashio et al., 2009), N-acetyl-L-cysteine in the C57BL/6J mouse strain (Davis et al., 2007) or a combination of vitamins A, C, and E, L-carnitine and α-lipoic-enriched diet in CBA/J mice (Sha et al., 2012) did not improve auditory function or reduce ARHL. Negative results are of course more difficult to interpret. They may be due to inadequate dosing or to species differences or to the different experimental conditions used for the administration of antioxidants. Also, targeting a single factor responsible for the pathogenesis of the ARHL may not be sufficient to avoid or reduce the effects of aging on hearing.

Given the fact that strial presbyacusis could be at the origin of most forms of ARHL (Schuknecht and Gacek, 1993; Gates and Mills, 2005; Schmiedt, 2010; Clinkard et al., 2013; Lee, 2013), and that this pathology is caused at least in part by alterations in the microvasculature and decreased strial blood flow, the use of cochlear vasodilators to improve auditory thresholds during aging seems a reasonable option. Indirectly supporting this idea, the administration of hydrogen sulfide as a vasodilator following noise-induced hearing loss has been proven to have a protective effect on the inner ear as it reduces cochlear damage and improves auditory function (Li et al., 2011). A similar positive response on hearing was observed when using hydroxyethyl starch but not when pentoxifylline was administered in guinea pigs after noise trauma (Lamm and Arnold, 2000). As opposed to these latter findings, a recent study in guinea pigs concluded that the administration of pentoxifylline after noise overexposure produced a near-normal auditory brainstem response and reduced the damage to the organ of Corti (Kansu et al., 2011). Another potential cochlear vasodilator that has been used as otoprotector is Mg^2+^ (Figure 2C). Pharmacological properties of this cation include increased cochlear blood flow (Haupt and Scheibe, 2002), modulation of the NMDA glutamate receptor, regulation of influx of calcium into the sensory hair cells and also calcium channel permeability (Günther et al., 1989; Cevette et al., 2003; Le Prell et al., 2007b). The effectiveness of magnesium in protecting the cochlea from noise insult has been shown in guinea pigs (Ising et al., 1982; Scheibe et al., 2000; Miller et al., 2003; Le Prell et al., 2007a), CBA/J mice (Le Prell et al., 2011) and humans (Attias et al., 1994). Of relevance, magnesium has a greater otoprotective effect after noise trauma in CBA/J mice when coupled to free radical scavengers than that observed when either magnesium or antioxidants are used individually as micronutrients (Le Prell et al., 2007a). Indeed together their protective effect is significantly greater than the sum of the individual agent protective effects. A similar combination of micronutrients has been demonstrated to reduce gentamicin-induced ototoxicity, reducing the threshold shift for frequencies at 12 kHz and below and protecting inner and outer hair cells in the upper half of the cochlea (Le Prell et al., 2014). As presbyacusis shares physiopathological alterations with noise and drug induced hearing loss, vasodilators like Mg^2+^ may protect the inner ear during aging (Figure 2C). Nonetheless, despite the benefits of using vasodilators for the treatment of hearing loss, as described in this review, there are no studies to date that have assessed its effects either individually or in combination on ARHL.

## Conclusions

In the light of evidence presented in this review, focused on the key roles of free radicals and reduced blood flow in pathogenesis of stress-induced hearing loss, we propose that a combined therapy targeting these specific factors, which are well implicated in the genesis and/or progression of presbyacusis, may attenuate ear damage and therefore, improve auditory function during aging. While there is not yet an effective medication to prevent a multifactorial and complex pathological condition such as ARHL, a treatment based on the synergistic effects of natural micronutrients such as the antioxidants vitamins A, C and E and the vasodilator magnesium all with good safety profiles, seems to be an excellent and promising efficacious therapeutic alternative for the treatment of this sensory impairment associated with aging.

## Author and Contributors

Drafting of the manuscript: JCA and VF-S. Design of figures: JCA, VF-S, PM-R, MCG-U. Critical revision of the manuscript for important intellectual content: JCA, VF-S, PM-R, MLV, JMM and JMJ.

## Conflict of Interest Statement

The authors declare that the research was conducted in the absence of any commercial or financial relationships that could be construed as a potential conflict of interest.
